# Physical activity interventions to improve cognition in first-episode psychosis: What we know so far

**DOI:** 10.1192/j.eurpsy.2024.1792

**Published:** 2024-12-16

**Authors:** Cinzia Perlini, Maria Gloria Rossetti, Francesca Girelli, Marcella Bellani

**Affiliations:** 1 Section of Clinical Psychology, Department of Neurosciences, Biomedicine and Movement Sciences, University of Verona, Verona, Italy; 2 Section of Psychiatry, Department of Neurosciences, Biomedicine and Movement Sciences, University of Verona, Verona, Italy; 3 Unit of Psychiatry, Azienda Ospedaliera Universitaria Integrata (AOUI) Verona, Verona, Italy; 4 Department of Neurosciences and Mental Health, Fondazione IRCCS Ca’ Granda Ospedale Maggiore Policlinico, Milan, Italy

**Keywords:** aerobic exercise, cognition, first-episode psychosis, physical activity

## Abstract

**Background:**

Cognitive impairment is a core feature of psychosis, which adversely affects global functioning and quality of life and has been consistently reported from the early stages of illness. Patients with first-episode psychosis (FEP) exhibit deficits in processing speed, short-term memory, attention, working memory, and executive functioning, which respond poorly to psychotropic drugs. Among non-pharmacological approaches, physical activity has shown promise in improving cognitive functioning in schizophrenia spectrum disorders. However, current evidence lacks specific data on individuals with FEP. In this review, we aim to explore the potential role of physical activity-based interventions in ameliorating the cognitive functions of people with FEP.

**Methods:**

The literature search was conducted on PubMed, PsycINFO, and Web of Science in March 2024, identifying 127 de-duplicated records. One additional article was identified by screening the reference lists of the included studies. A total of six studies fulfilled the inclusion criteria and were reviewed. They all analyzed the effect of structured physical activity interventions on the cognitive functioning of patients with FEP.

**Results:**

Preliminary findings suggest that physical activity interventions enhance memory, attention, and executive functions of patients with FEP but not social cognition and motor function.

**Conclusions:**

Study differences in sample characteristics, design, and intervention protocols prevent firm conclusions about the cognitive-boosting effects of the interventions in FEP. Further studies using more rigorous methodologies are needed to understand the durability of these effects and the underlying mechanisms.

## 1. Introduction

Cognitive deficits are a core feature of schizophrenia spectrum disorders, which manifest from the onset of the disease and persist over time [1]. These impairments affect multiple domains, including working memory, attention, social cognition, processing speed, verbal memory, visual memory, and problem-solving, and contribute to poor functional outcomes [2]. Notably, attention and working memory are strong predictors of functional recovery following a first episode of psychosis (FEP) [3]. Moreover, social cognition has been shown to predict real-world functioning more accurately than neurocognitive performance [4]. Thus, implementing interventions targeting cognition from the early stages of illness may facilitate functional recovery. In 2022, the European Psychiatric Association (EPA) published evidence-based guidelines for the treatment of cognitive impairment in schizophrenia spectrum disorders, [5] and, among psychosocial interventions, physical exercise was recommended as an adjunctive treatment to improve symptoms, cognitive performance, and quality of life [5, 6]. Recent meta-analyses have shown that aerobic exercise has small-to-moderate positive effects on global cognition, working memory, and attention/vigilance, while no improvements were observed in visual learning or reasoning and problem-solving [7, 8]. Conversely, the results for processing speed, verbal memory, and social cognition were mixed, likely due to differences in statistical analysis methods [7, 8]. Interestingly, interventions were more effective in improving cognition when they involved a greater amount of exercise, took place in a group setting, and were supervised by physical activity professionals [7, 8].

The current EPA guidelines are primarily based on meta-analyses involving patients in the chronic phase of the disorder [5]. Furthermore, to date, only one systematic review has explored the effect of physical activity-based interventions in FEP populations, reporting a paucity of available studies, most of which have focused on clinical rather than cognitive outcomes [9]. As a result, whether physical activity interventions have a cognitive-enhancing effect on FEP remains to be clarified.

To fill this literature gap, the present systematic review aims to summarize and critically appraise evidence on the potential role of physical activity-based interventions in improving the cognitive functioning of patients with FEP. Based on findings in chronic populations, we expect these interventions to have small-to-moderate positive effects on global cognition, working memory, and attention, at least.

## 2. Methods

The literature search, screening, and selection of the studies were reported according to the Preferred Reporting Items for Systematic Reviews and Meta-Analyses (PRISMA) guidelines [10].

### 2.1. Literature search

The data search was performed on 7 March 2024 using PubMed, Web of Science, and PsycINFO databases. The following keywords were used for the search: (“First episode psychosis” OR “FEP” OR “early psychosis”) AND (“physical activity” OR “physical exercise” OR “aerobic exercise” OR “resistance training” OR “exercise therapy”) AND (cognit* OR neurocognit* OR “executive function*” OR learning OR memory OR attention).

### 2.2. Inclusion and exclusion criteria

Inclusion and exclusion criteria were based on the Population, Intervention, Comparison, Outcomes and Study (PICOS) design framework [11].

Inclusion criteria: studies that (i) involved patients diagnosed with FEP, as defined by each study protocol; (ii) utilized structured physical activity interventions as part of the treatment or management of FEP (iii) may or may not include a comparison group (e.g., a control group receiving standard care or no intervention); (iv) measure cognitive performance using standardized neuropsychological tests; (v) had a pre-post intervention study design (RCTs or quasi-experimental studies).

Exclusion criteria: articles not written in English; non-peer-reviewed, unpublished, or not empirical studies (e.g., dissertations, conference abstracts, book chapters, case reports, reviews, and meta-analyses).

### 2.3. Data screening

The studies were screened using the Rayyan web app [12]. MGR and FG conducted the screening independently, and any ambiguities about the inclusion of the studies were resolved through discussion with CP. Studies were first screened against exclusion and inclusion criteria using titles and abstracts. Next, full-text articles were assessed for eligibility in the systematic review. Finally, the reference lists of the included studies were examined to identify any additional studies that met the inclusion criteria for this review.

### 2.4. Data extraction

FG and MGR carried out data extraction. Information was gathered from tables, figures, and written summaries of each study and compiled into two tables. Table 1 shows details on publication characteristics (first author, year, study design); sample characteristics (sample size, diagnosis-related inclusion criteria, sex, age, medications); characteristics of physical activity-based interventions (protocol, duration, frequency, overall length, setting, adherence); control group details; dropout rate; and cognitive outcome measures. Table 2 provides an overview of the tests-subtests used in each study to assess cognition, along with the effect sizes for pre-post treatment differences in cognitive performance.

**Table 1.** Characteristics of the included studies

Author, year	Study design	Sample size (% male)	Diagnosis-related inclusion criteria	Age	Medications	Physical activity-based intervention				Control group	Dropout rate	Cognitive outcome measures
						Type and length of activity	Frequency and overall duration	Setting and format	Attendance rate			
Lin et al., 2015 [19]	Single-blind RCT	124 (0)	Being a current service user of EASY^a^ service of Hong Kong, within the first 5 yrs from the onset of the psychotic disorder	24.6 (7.6)	94% of pz received 1^st^–2^nd^ generation antipsychotics as monotherapy (n = 109) or polypharmacy (n = 8)	Aerobic exercise; ≥ 60 min/session	3 times/w for 12 weeks	Clinic (group)	AE: 47%	YG WL (TAU)	YG: 24% AE: 27% WL: 18%	HKLLT, Digit span test, LCT, Stoop test
Nuechterlein et al., 2016 [17]	Non-randomized controlled feasibility study	16 (73)	A recent onset of psychotic illness, with the beginning of the first major psychotic episode within the last 2 yrs	22.7 (4.7)	100% of pz received treatment with a 2^nd^ generation antipsychotic	Aerobic exercise; 45 min (clinic) + 30 min (home)/session.	2 times/w for 10 weeks	Clinic and home (individual/group)	Clinic: 95% Home: 92%	CT: 2 h 2 times/w (perceptual processing and verbal learning and memory)	CT: 0% CT + PA: 0%	MCCB
Firth et al., 2018a [15]	Non-randomized controlled feasibility study	31 (81)	Being a current user of the EI^b^ services within the first 5 yrs of onset of a psychotic disorder	25.8 (4.6)	100% of pz were on antipsychotic medications	Moderate-to-vigorous aerobic/anaerobic exercise; (≥ 90 min/session).	2 times/w for 10 weeks	Clinic and/or home (individual/group)	≥90 min/w (50%) ≥60 min/w (79%)	TAU from another trial^a^	EG: 19%	CANTAB, TMT, Erikson Flanker Task, Motor screening, Mind in the eyes
Firth et al., 2018b [16]	Non-randomized single-arm feasibility study	28 (ns)	Being a current user of EI^b^ services within the first 5 yrs of a psychotic disorder	27. (4.5)	100% of pz were taking antipsychotic medications	Moderate-to-vigorous aerobic/anaerobic exercise; ≥ 90 min/session.	2 times/w for 10 weeks	Clinic and/or home (individual/group)	55% (11 of 20) continued to exercise weekly; 45% (9 of 20) participants exercised less than 1 time/w or not at all.	na	15%	CANTAB, TMT Erikson Flanker Task, Motor screening, Mind in the eyes
Hallgren et al., 2019 [18]	Non-randomized single-arm pre-post study	63 (64)	Young adults (18–45 yrs) with FEP as defined by the ICD–10.	30 (7.6); 29 (6.9)	ns	Aerobic/anaerobic exercise (circuit training); 60 min/session.	3–5 times/w per 12 weeks	Clinic (group)	No exercise: 22%; ⩾12 sessions: 48%; 1–11 sessions: 30%	na	32%	CBB, TMT
Suen et al., 2023 [20]	Non-randomized single-arm pre- post study	49 (12)	People with a primary diagnosis of schizophrenia spectrum disorder referred by the EASY^a^ service of Hong Kong or self-referred via web	38.1 (13.2)	ns	Static/dynamic exercises and yoga postures; 90 min/session.	1 time/w per 12 weeks	Community locations (group)	ns	na	22%	Digit span WAIS-R subtest

Age and time in early intervention services are expressed as mean (sd).

AE = Aerobic Exercise; BD = Bipolar Disorder; CANTAB = Cambridge Neuropsychological Test Automated Battery; CBB = Cogstate Brief Battery; CT = Cognitive Training; d = day; EG = exercise group; EIS = Early Intervention Services; FEP = First Episode Psychosis; h = hour; HKLLT = Hong Kong List Learning Test; LCT = Letter Cancellation Test; MCCB = MATRICS Consensus Cognitive Battery; m = months; min = minutes; na = not applicable; ns = not specified; PA = Physical Activity; PT = Personal Trainer; Pz = patients; SCZ = Schizophrenia; STM = short-term memory; TAU = Treatment As Usual; TMT = Trail Making Test; w = week; WAIS-R = Wechsler Adult Intelligence Scale-Revised; WL = Waiting List; WM = Working Memory; yrs = years; YG = Yoga group.

a EASY = Early Assessment Service for Young People with Psychosis. EASY is offered to people with psychotic symptoms aged 15–25 years, who did not already receive treatment for psychosis.

b EI = Early Intervention. In the UK, the EI services are offered to individuals aged 14–35 who are experiencing FEP (defined as full-threshold psychotic symptoms for a period of greater than 7 consecutive days, regardless of formal diagnostic status).

**Table 2.** Pre-post treatment cognitive changes associated with physical activity-based interventions in first-episode psychosis

Cognitive function	Test/subtest	Performance	Effect size
**General cognitive ability**			
Nuechterlein et al. (2016) [17]	MCCB/composite	↑	*f* = 0.48^a^
**Memory**			
**Verbal memory**			
Lin et al. (2015) [19]	HKLLT/verbal acquisition	↑	*d* = 0.83
	HKLLT/verbal retention	↑	*d* = 0.56
	Digit span forward	↑	*d* = 0.59
Nuechterlein et al. (2016) [17]	MCCB/verbal learning	–	ns
Firth et al. (2018a) [15]	CANTAB/verbal STM	↑	*d* = −0.88
Firth et al. (2018b) [16]	CANTAB/verbal STM	↓	ns
Suen et al. (2023) [20]	Digit span forward	↑	*d/r_b* = 0.04
**Visual memory**			
Hallgren et al. (2019) [18]	CBB/visual learning	↑	*g* = 0.47
Firth et al. (2018a) [15]	Spatial span	–	*d* = −0.07
	Digit coding	↑	*d* = −0.17
Nuechterlein et al. (2016) [17]	MCCB/visual learning	–	ns
**Working memory**			
Lin et al. (2015) [19]	Digit span backward	↑	*d* = 1.08
Nuechterlein et al. (2016) [17]	MCCB/WM	↑	*f* = 0.50^a^
Hallgren et al. (2019) [18]	CBB/WM	↑	ns
Suen et al. (2023) [20]	Digit span backward	↑	*d/r_b* = 0.18
**Executive functions**			
**Attention**			
Lin et al. (2015) [19]	LCT/attention	↑	*d* = 0.22
Nuechterlein et al. (2016) [17]	MCCB/attention, vigilance	↑	*f* = 0.33^a^
Hallgren et al. (2019) [18]	CBB/attention	–	ns
**Processing speed**			
Nuechterlein et al. (2016) [17]	MCCB/ processing speed	↑	*f* = 0.38^a^
Firth et al. (2018a) [15]	TMT-A	↑	*d* = 0.40
	TMT-B	↑	*d* = 0.65
Firth et al. (2018b) [16]	TMT-A	↑	ns
	TMT-B	↑	ns
Hallgren et al. (2019) [18]	CBB/ processing speed	↑	*g* = 0.49
	TMT-A	↑	*g* = 0.48
	TMT-B	↑	ns
**Inhibitory control**			
Lin et al. (2015) [19]	Stroop test congruent condition, correct items	↑	*d* = 0.30
	Stroop test incongruent condition, correct items	↑	*d* = 0.36
Firth et al. (2018a) [15]	Flanker conflict cost – accuracy	↑	*d* = 0.53
	Flanker conflict cost – time	↓	*d* = −0.05
Firth et al. (2018b) [16]	Flanker conflict cost – accuracy	↑	ns
	Flanker conflict cost – time	↓	ns
Firth et al. (2018a) [15]	Stockings of Cambridge	↑	*d* = −0.48
Firth et al. (2018b) [16]	Stockings of Cambridge	↑	ns
**Social cognition**			
Nuechterlein et al. (2016) [17]	MCCB/social cognition	↑	*f* = 0.65^a^
Firth et al. (2018a) [15]	Mind in the eyes	↑	*d* = −0.67
Firth et al. (2018b) [16]	Mind in the eyes	–	ns
**MOTOR FUNCTIONING**			
Firth et al. (2018a) [15]	Motor screening	↓	*d* = −0.06
	Finger tapping	↑	*d* = −0.12

CANTAB = Cambridge Neuropsychological Test Automated Battery; CBB = Cogstate Brief Battery; *d* = Cohen’s *d*; *f* = Cohen’s *f*; FEP = First Episode Psychosis; *g* = Hedges’ *g;* HKLLT = Hong Kong List Learning Test; LCT = Letter Cancellation Test; MCCB = MATRICS Consensus Cognitive Battery; ns = not specified; *r_b*, Rank-biserial correlation effect size; STM = short-term memory; TMT(A/B) = Trail Making Test (part A and B).

↑ = Better post *vs* pretreatment performance.

↓ = Worse post *vs* pretreatment performance.

– = No differences in pre-post treatment performance.

a Aerobic exercise combined with cognitive training group *vs* cognitive training group.

### 2.5. Risk of bias assessment

MGR and FG assessed the risk of bias in the reviewed literature via the Joanna Briggs Institute (JBI) critical appraisal tools for randomized controlled trials [13] and quasi-experimental studies [14]. These tools evaluate the extent to which a study has addressed the possibility of bias in its design, conduct, and analysis (yes, no, unclear, not applicable). Disagreements in the evaluation were solved by discussion until a consensus was reached.

## 3. Results

### 3.1. Included studies

**Figure 1.**
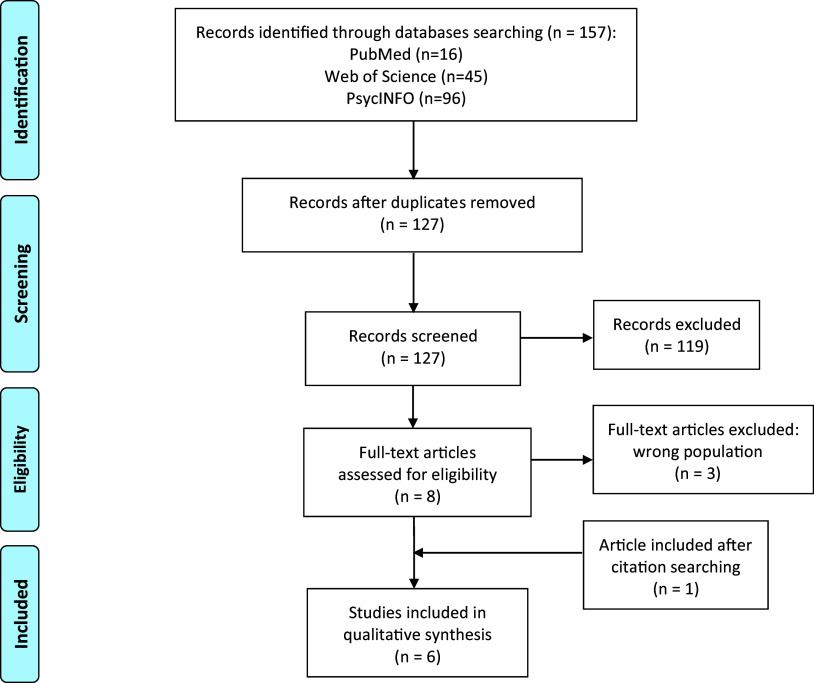
PRISMA flow diagram of systematic search and study selection process.

The literature search yielded 127 de-duplicated records. After screening titles and abstracts, 119 articles were excluded for not meeting the inclusion criteria. Eight articles were selected for full-text review, after which five studies were included. An additional study was identified by examining the reference lists of the included studies. In total, six studies were included in the review. A flowchart illustrating the studies’ selection process is displayed in Figure 1.

### 3.2. Risk of bias within studies

Details on the risk of bias assessments are given in Table S1. Overall, the included studies had a moderate risk of bias. The areas of greatest concern were (i) *selection and allocation*, as most studies did not have a control group, and (ii) *assessment, detection, and measurement of outcome*, as none of the studies reported information about numbers and training of outcome raters and intra/interrater reliability. Additionally, due to the lack of control groups, we could not assess the risk of bias related to other exposures/treatments occurring currently with the intervention of interest (*administration of intervention/exposure)* and whether or not loss to follow-up was related to characteristics of the intervention of interest *(participant retention).*

### 3.3. Studies synthesis

Study characteristics and intervention details are displayed in Table 1. The total number of participants ranged from 28 to 124, and the mean age was 23.5 years. Firth and colleagues used nearly overlapping samples [15, 16]. Half of the studies included mainly male participants [15, 17, 18]; two studies enrolled only [19] or mostly female participants [20], while the remaining study did not specify the sex distribution of the sample [16]. In two-thirds of the studies, patients with FEP were recruited through community-based early intervention services for psychosis [15, 16, 19, 20]. The mean years since first contact with these services or generic psychiatric services ranged from 0.75 to 3.6. Finally, four out of six studies reported data on medications showing that almost all patients were taking antipsychotics [15–17, 19].

### 3.4. Characteristics of physical activity-based interventions

All studies but two [15, 16] used different protocols for the physical activity-based intervention. Patients recruited by Hallgren et al. were asked to practice aerobic exercise 1 h/day, 3–5 times per week, for 12 weeks. The circuit training provided: 5′–10’ warm-up; 45′ high-volume resistance exercises; and 5′-10′ cool-down/stretch. Participants were invited to reach a subjectively moderate intensity and received a motivational intervention to maintain their adherence to the program [18]. Firth and colleagues tailored the exercises to individual preferences to keep treatment motivation high. In particular, participants were asked to do at least 90 minutes of moderate-to-vigorous activity twice per week for ten weeks [15, 16]. The gym training included aerobic exercise (treadmills, cycle ergometers, and rowing machines) and resistance activities (exercises to increase muscle tone and strength). To reach the expected weekly amount of physical activity, the participants could carry out other sporting activities (i.e., football, boxing/martial arts, cycling, badminton, swimming).

Nuechtelein et al. structured an incentive reward program aimed at preventing motivational decline. The program consisted of practicing aerobic exercise at the clinic (45 minutes) and at home (30 minutes) twice a week while watching aerobic exercise videos involving calisthenics and simple movement sequences. The intensity was individually adjusted at 60–80% of aerobic capacity (Karvonen formula [21]). Patients received a financial reward for every homework session completed. The amount gained also varied according to achieving specific goals during the sessions at the clinic [17]. Lin and colleagues proposed a 60-minute session of aerobic exercise (i.e., walking and cycling) three times a week for 12 weeks. The active control group underwent 12 weeks of yoga exercise [19]. Lastly, the 12-week community exercise program named FITMIND consisted of 12 weekly 90-minute sessions conducted by trained volunteers [20]. Specifically, the protocol was tailored to patients with psychosis and included ten static and dynamic exercises and 23 yoga “easy-to-learn” postures.

Regarding the intervention format, all studies included group sessions [14–18], while three of them also included individual sessions [18–20].

Finally, in all but one study, physical activity was supervised by professional trainers (i.e., research assistants with exercise experience, trained staff members, and certified coaches) [15–19].

### 3.5. Feasibility of physical activity-based interventions

All studies reported attrition and dropout rates. The mean attrition rate was approximately 26%, while the mean dropout rate was ~17%. Common causes of attrition (other than voluntary dropout) included loss of contact with psychiatric services, hospitalization due to mental health illness, and missing data, while dropout was often attributed to lack of motivation or time, long-distance travel, and physical illness. Exercise adherence rates were heterogeneous, ranging from 30% [18] to 95% [17] of total sessions. Two out of six studies reported on the safety of the intervention, showing no side effects attributable to the physical exercise [18, 19].

### 3.6. Posttreatment cognitive response

Table 2 shows posttreatment changes in the cognitive functioning of FEP treated with psychical activity-based interventions. The identified studies investigated a variety of cognitive domains, including verbal, visual and working memory; attention and task-shifting; speed of processing; inhibitory control; reasoning and problem-solving; social cognition; and motor functioning. Short-term verbal memory significantly improved after aerobic exercise programs delivered alone [15, 19] or combined with yoga [20]. In some cases, these gains were temporary, reverting within six months [16]. Long-term verbal memory, however, showed no significant changes [17, 19]. Aerobic circuit training enhanced long-term visual memory, while combined cognitive and aerobic exercise did not have the same effect [17, 18]. Short-term visual memory remained largely unaffected by aerobic exercise [15]. Aerobic exercise programs alone [19, 20] or combined with cognitive training [16] ameliorated also working memory.

Regarding executive functions, findings showed that cognitive training paired with aerobic exercise improved sustained attention [17]. Similarly, aerobic training involving walking and cycling improved selective attention [19], while aerobic circuit training did not [18]. Firth and colleagues found that aerobic exercise enhanced processing speed, with gains lasting up to 6 months [15, 16]. Hallgren’s study partially confirmed these improvements [18]. Postintervention processing speed also increased when measured with Cogstate Brief Battery (CBB) and MATRICS Consensus Cognitive Battery (MCCB) [17, 18].

Results on inhibitory control were mixed. Firth et al. observed improvements in inhibitory control after aerobic exercise, but these effects disappeared after six months [15, 16]. Conversely, Lin et al. found no improvement in inhibitory control following aerobic exercise [19]. Physical activity enhanced reasoning and problem-solving [18].

Social cognition and motor function were little investigated, and none of the studies detected significant changes after the physical activity program [15–17].

Additionally, findings from Nuechterlein et al. showed that physical exercise might boost the impact of cognitive training on global cognition [17].

Interestingly, three authors reported possible dose–response associations between physical activity and cognitive outcomes. Firth et al. found that the amount of exercise achieved was significantly correlated with improvements in processing speed [15]. Lin and colleagues found a positive correlation between the number of sessions attended and the posttreatment working memory score [19]. Hallgren’s study noted that the total training frequency was significantly correlated with visual attention performance of male participants. Nonetheless, the duration of the exercise intervention and the number of sessions per week were not related to the intervention’s effectiveness [18].

Finally, two studies showed that social support may facilitate patients’ motivation and adherence to physical activity [16, 20].

## 4. Discussion

This systematic review aimed to investigate the impact of physical activity-based intervention on cognitive functioning of people with FEP. Six studies were eligible, most of which used group-based aerobic exercise interventions conducted by trained professionals. Overall, the findings confirmed our hypothesis, showing that physical activity-based interventions may have small-to-large positive effects on global cognition [17], verbal and working memory [15, 17–20], attention [17, 19], processing speed [15–18], and social cognition [15, 17] of patients with FEP. Nevertheless, the maintenance of the effects over time remains to be determined. Conversely, mixed or negative effects were observed when measuring the impact of the interventions on motor functioning [15] and inhibitory control [15–17, 19]. Inconsistencies of results may be due to differences between intervention protocols and outcome measures.

Our findings align with previous meta-analytic evidence showing positive effects of aerobic exercise on cognitive functioning in schizophrenia spectrum disorders [7, 8]. Interestingly, we noted that the effect sizes reported in single studies on FEP were mainly medium to large and speculated that, similar to other cognitive enhancement interventions [22], physical activity might confer greater cognitive benefits in the early rather than chronic phase of the illness. However, the factors associated with a possible higher level of responsiveness in FEP remain to be clarified. One reason might be the specific characteristics of the exercise-based interventions carried out in the reviewed studies, which were delivered in group sessions and led mainly by exercise professionals. Notably, both group-based format and professional guidance have been suggested as factors that enhance the effectiveness of such interventions [7, 8]. Other factors associated with intervention effectiveness were exercise dosage (intensity, frequency) and social support by peers and/or exercise trainers. Evidence of a dose–response relationship between exercise and neurocognitive performance has been previously reported in people with schizophrenia [7, 8], as well as a positive association between group-based training/team sports, social support, and exercise adherence [8, 23]. Social support can boost intrinsic motivation [24] which, in turn, enhances cognition [8, 25]. Moreover, it can be speculated that greater adherence and effectiveness of group *versus* individual interventions may be due to the increased interpersonal coordination and social interaction demands of the former, as well as to the involvement of higher cognitive functions like executive functioning and attention. This has been previously observed for team sports in schizophrenia [26, 27].

Finally, the acceptability of the exercise-based interventions in the current review was good and comparable to those reported for other psychosocial interventions specifically tailored to enhance cognition (that is cognitive remediation training) [28, 29]. Specifically, the average dropout rate was ~15% and did not differ from those in the control intervention when available.

Most of the reviewed studies speculated that physical exercise might exert a beneficial effect on cognition via neuroplasticity-based mechanisms (i.e., increment of cortical blood flow, modulation of synaptic connections, and neurogenesis) [15–19]. This hypothesis is corroborated by evidence showing that physical activity, mainly aerobic exercise, is associated with morphofunctional changes in the brain of healthy populations [30] and people with early onset/chronic schizophrenia [19, 31]. However, the cellular processes through which exercises increase neurogenesis and cognitive performance remain to be disentangled. Alternatively, it has been suggested that physical activity might improve cognition indirectly since it ameliorates clinical symptoms and global and social functioning of people with schizophrenia spectrum disorders, all of which are associated with cognitive functioning [7].

Some limitations should be considered when interpreting the results. Most studies lack a randomized controlled design (control group or control intervention), making it difficult to determine if the observed effects are directly attributable to physical activity interventions. Moreover, the small sample sizes, except in Lin’s study [19], further limit statistical power and increase the risk of Type II errors.

Additionally, the inconsistent criteria used to define FEP, along with varying patient characteristics such as gender ratios, medications, and dropout rates, hinder the generalization of results. Furthermore, differences in the protocols of the physical activity interventions (e.g., frequency, setting, duration, and intensity) also mine the comparisons among studies. Finally, differences in the effects of exercise across specific cognitive subdomains could be attributable to the variety of tests used to measure cognitive outcomes in FEP.

## 5. Conclusion

The available evidence is promising and suggests that physical activity-based interventions positively affect the cognitive functioning of people with FEP, particularly within verbal and working memory, attention, processing speed, and social cognition domains. In line with previous evidence on schizophrenia spectrum disorders, a higher load of exercise, the group format, and the presence of social support are key factors associated with the effectiveness of exercise-based interventions. Future studies applying more rigorous and homogeneous methodologies are needed to explore the effectiveness of the interventions and the mechanisms behind exercise-induced cognitive improvements. Furthermore, future studies should compare people with first episode *versus* chronic psychosis to verify the hypothesis that exercise-based interventions produce larger cognitive improvements if administered in the very early phase of the disease. Notably, physical exercise is the only evidence-based psychosocial intervention, besides cognitive remediation training, that targets cognitive function and has been recommended for treating cognitive impairment in schizophrenia spectrum disorders [5, 28]. Therefore, future research should also focus on exploring the feasibility and usability of exercise-based interventions in clinical practice, as implementing such interventions from the very early stages of the disease may facilitate the functional recovery of people with schizophrenia spectrum disorders.

## Supporting information

Perlini et al. supplementary material Perlini et al. supplementary material

## Data Availability

This review did not generate any new data. All data included in this review are from publicly available studies, which are referenced in the manuscript.

## Supplementary material

The supplementary material for this article can be found at http://doi.org/10.1192/j.eurpsy.2024.1792.
